# Association of cardiovascular health at old age with all-cause mortality: a prospective cohort study in China

**DOI:** 10.1186/s12877-023-04093-9

**Published:** 2023-07-15

**Authors:** Shimin Chen, Haowei Li, Shengshu Wang, Shanshan Yang, Shaohua Liu, Yang Song, Xuehang Li, Rongrong Li, Jianhua Wang, Miao Liu, Yao He

**Affiliations:** 1 Institute of Geriatrics, Beijing Key Laboratory of Aging and Geriatrics, National Clinical Research Center for Geriatric Disease, Second Medical Center of Chinese PLA General Hospital & Chinese PLA Medical School, No.28 Fuxing Road, Haidian District, Beijing, 100853 China; 2 Department of Disease Prevention and Control, First Medical Center, Chinese PLA General Hospital & Chinese PLA Medical School, Beijing, China; 3 Department of Statistics and Epidemiology, Graduate School, Chinese PLA General Hospital & Chinese PLA Medical School, No.28 Fuxing Road, Haidian District, Beijing, 100853 China

**Keywords:** Cardiovascular health, All-cause mortality, Elderly, Cohort study

## Abstract

**Background:**

Cardiovascular disease has become the leading cause of death worldwide, but there is a lack of data on whether cardiovascular health (CVH) is associated with elderly mortality in China. We investigated the relationship between the ideal CVH score of Chinese elderly and the all-cause mortality.

**Methods:**

The Beijing Elderly Comprehensive Health Cohort Study included a total of 4,499 participants aged 60 years and above. The CVH metric was calculated at baseline and had a score ranging from 0 to 12. The relationship of CVH metrics with all-cause mortality was investigated using Cox proportional hazard regression analysis. The robustness of results was tested using subgroup and sensitivity analysis.

**Results:**

The median CVH score among participants was 8.00 (2.00), with only 8.0% scoring 11–12 points. 667 deaths were observed during an average follow-up time of 8.2 years. Participants with a CVH score of 11–12 had a decreased risk of all-cause mortality when compared to those with a CVH score of 0–4(HR = 0.584, 95% CI: 0.373–0.913). Participants had a 7.5% lower risk of all-cause death with each unit higher CVH score (HR = 0.925, 95%CI: 0.885–0.967) with a linearly decreasing trend (P _nonlinear_ = 0.575). The relationships were greater in younger elderly people and stroke patients (P _interaction_ = 0.011 and 0.037. respectively). The consistency of significant trends in sensitivity analysis shows the robustness of association (P _trend_ < 0.001).

**Conclusions:**

Among the Chinese elderly, there was a linear relationship between improving CVH scores and a lower risk of all-cause mortality. Because of the enormous benefits brought by one point, strategies are essential for improving cardiovascular health attainment.

**Trial registration:**

This study was registered at China Clinical Trial Registration Center (ChiCTR2100049866).

**Supplementary Information:**

The online version contains supplementary material available at 10.1186/s12877-023-04093-9.

## Background

Cardiovascular disease (CVD) continues to be the leading cause of death and rising healthcare costs worldwide [1]. It increased the number of deaths from 12.1 million in 1990 to 18.6 million in 2019 [2], and this number is still increasing steadily [3]. Every year, nearly 4 million people in China died from CVD [4]. The number of prevalent cases reached 94 million in 2016, more than doubling that of 1990 [5]. With rapid aging, the number of people aged 65 and above will reach 366 million (26.1%) by 2050 [6], with a significant shift toward CVD in old age [7]. CVD events are expected to increase by 50% per year from 2010 to 2030 due to aging, and by 23% based on the current risk factor trend [8, 9]. The excess burden of CVD among the elderly in China poses a significant challenge to the medical and health care systems [10]. Despite advances in the medical system and health institutions in the treatment and care of CVD, the significant burden of cardiovascular disease risk factors in China continues to rise [11].

The American Heart Association (AHA) released 2020 Impact Goals in 2010 to emphasize the importance of public cardiovascular health prevention. It also defined the concept of Ideal Cardiovascular Health (ICH), also known as the Life's Simple 7 principles [12]. Since then, CVH has been widely adopted in numerous studies, aiming at promoting the interventions of modifiable health behaviors and CVD risk factors [13–18]. Increasingly, studies show that having a higher level of CVH can reduce the risk of various chronic diseases (CVD events [19–21], caners [22, 23], cognitive impairment [24–26], and so on) and mortality [27–29].

We conducted a thorough literature review prior to beginning this research. From inception to 2022/07/07, PubMed, Web of Science, and Embase were used to conduct searches. The following keywords were applied: (“ideal cardiovascular health” OR “cardiovascular health metrics” OR “life’s simple 7”) AND (“mortality” OR “death”). The reference lists of eligible literature were thoroughly searched. The effect of CVH on mortality risk has been studied in younger Chinese people [30–33]: the HRs ranged from 0.54 to 0.78 (highest CVH group vs. lowest CVH group), but data for the elderly in China is limited. In view of this situation, In light of this, we intend to examine the relationship between CVH score and all-cause mortality in the elderly in China from 2009 to 2021 using data from the Beijing Elderly Comprehensive Health Cohort Study (BECHCS).

## Methods

### Study design and population

BECHCS was a community-based cohort study conducted in Beijing. As shown in Supplemental Fig. 1, the survey areas include Wanshou Road Community and Miyun District, which were chosen as representatives of the geographical and economic features of Beijing, China. A two-stage random cluster sampling method was used to recruit qualified subjects (aged 60 years and above, who had lived in the local areas for at least 12 months).

The baseline survey was carried out from September, 2009 to September, 2014, which included standardized questionnaires, physical examinations and laboratory tests. And follow-up data have been reviewed until 31^st^ March, 2021 in order to collect information on mortality. We eventually included 4,499 participants after excluding those who did not follow up, had incomplete information, or had missing values.

All the experiment protocol involving human beings are in line with the guiding principles of national, international, institutional, and Declaration of Helsinki in the manuscript. The BECHCS program was approved by the Independent Ethics Committees of Chinese People’s Liberation Army General Hospital(S2021-327–01). This study was registered at China Clinical Trial Registration Center (ChiCTR2100049866). All data were anonymous to protect participants’ privacy in the research. Prior to the study, participants were given oral and written information about the study and signed informed consent forms.

### Metrics and score for CVH

According to the guidelines of AHA [12] and modified according to the characteristics of the elderly in China [34], 6 metrics (include: cigarette smoking, body mass index, physical activity, total cholesterol, blood pressure and fasting plasma glucose) were used to define and monitor the status of cardiovascular health (CVH). Interview questionnaires, physical examinations, and laboratory measurements were used to collect data for each metric. The cutoff points for poor, intermediate, and ideal status in each CVH metric are listed in Supplemental Table 1. Each behavior or factor was graded as poor (0 point), intermediate (1 point), or ideal (2 points) (2 points). The CVH score was calculated by adding the sum of six CVH metrics, which ranged from 0 to 12 points.

**Table 1:** Characteristics of study sample at baseline based on category of residence

Characteristic	Total	Urban	Rural	*P* value
Age, y	70.00(10.00)	72.00(9.00)	68.00(10.00)	< 0.001
Gender				0.974
Male	1814(40.3)	847(40.3)	967(40.3)	
Female	2685(59.7)	1255(59.7)	1430(59.7)	
Ethnic group				0.002
Han	4414(98.1)	2048(97.4)	2366(98.7)	
Others	85(1.9)	54(2.6)	31(1.3)	
Marital status				< 0.001
Married	1774(39.4)	1774(84.4)	0	
Single or widowed	2725(60.6)	328(15.6)	2397(100.0)	
Education, y				< 0.001
< 6	1199(26.7)	243(11.6)	956(39.9)	
6–11	2186(48.6)	774(36.8)	1412(58.9)	
> 11	1114(24.8)	1085(51.6)	29(1.2)	
Alcohol drinking				< 0.001
Never	2791(62.0)	1579(75.1)	1212(50.6)	
Former	250(5.6)	98(4.7)	152(6.3)	
Current	1458(32.4)	425(20.2)	1033(43.1)	
Coronary heart disease	934(20.8)	498(23.7)	436(18.2)	< 0.001
Hypertension	2427(53.9)	1513(72.0)	914(38.1)	< 0.001
Diabetes mellitus	601(13.4)	400(19.0)	201(8.4)	< 0.001
Stroke	602(13.4)	267(12.7)	335(14.0)	0.211
Dyslipidemia	793(17.6)	699(33.3)	94(3.9)	< 0.001
CVH^†^ metric				
Smoking status				< 0.001
Never	3127(69.5)	1531(72.8)	1596(66.6)	
Former	594(13.2)	340(16.2)	254(10.6)	
Current	778(17.3)	231(11.0)	547(22.8)	
Physical activity, h/wk				< 0.001
< 5	1780(39.6)	424(20.2)	1356(56.6)	
5–9	1726(38.4)	985(46.9)	741(30.9)	
> 9	993(22.1)	693(33.0)	300(12.5)	
Body mass index, kg/m^2^	24.44(4.40)	24.84(4.22)	24.33(4.41)	< 0.001
Systolic blood pressure, mmHg	134.00(24.00)	138.00(25.00)	134.00(24.00)	< 0.001
Diastolic blood pressure, mmHg	79.00(15.00)	77.00(14.00)	80.00(14.00)	< 0.001
Total cholesterol, mmol/L	4.89(1.34)	5.22(1.32)	4.62(1.25)	< 0.001
Fasting plasma glucose, mmol/L	5.55(1.15)	5.61(1.15)	5.49(1.13)	< 0.001

Median (IQR) and number (%) were summarized for continuous variables and categorical variables, respectively. Statistical significance for continuous variables was tested for Mann–Whitney U and χ^2^ method was conducted for categorical variables

^†^CVH indicates cardiovascular health

### Covariates

Data on demographic characteristics, medical histories, and lifestyle were gathered using standardized questionnaires administered in person by trained interviewers. Age, gender, residence, ethnic group, marital status, education, alcohol consumption, illness history, and so on. Physical examinations of participants were performed by experienced physicians and nurses in accordance with a standardized protocol, including weight, height, and blood pressure, among other things. Overnight blood sampling testing was used in the laboratory. Blood samples were sent in less than 30 min to the Chinese PLA general hospital’s central certified laboratory.

The primary outcome was all-cause mortality. The death was recorded by the Death Registration Center of Chinese Center for Disease Control and Prevention and confirmed by the public security department. The date, causes, and location of death were recorded with unique personal identification numbers based on death certificates.

### Statistical analysis

To describe the characteristics of the participants, mean (standard deviation) or median (interquartile range) values were summarized for normally distributed or non-normally distributed continuous variables, and as number (percentage) for categorical variables. Statistical significance for continuous variables was tested for Mann–Whitney U tests and χ^2^ method was conducted for categorical variables. We began by describing the distribution of each CVH metric by age, gender, and residential group. The percentage of each ideal CVH in each age and gender group was then depicted using a mosaic plot. The distribution of CVH scores by age, gender, and residence was depicted using percentage bar plots.

The second step was to use a restricted cubic spline with four knots to see if the relationship between CVH score and all-cause mortality fit the linear model. To confirm whether the CVH score meets the proportion hazard assumption, a survival curve with cumulative hazard was generated.

The third step was to use a Cox proportion regression model to estimate the hazard ratio (HR) and 95% confidence interval (95% CI) for the relationship between CVH score and all-cause mortality (crude, and controlling for age, gender, residence, ethnic group, marital status, education, alcohol drinking, previous coronary heart disease and stroke). Then the population attributable fraction (PAF) among CVH score groups was then calculated using the formula: $\mathrm{PAF}=\frac{\mathrm{pe}(\mathrm{HR}-1)}{1+\mathrm{pe}(\mathrm{HR}-1)}$, p_e_ refers to the proportion of exposed subjects in the population. The Mann–Kendall trend for mortality rate or density was used to calculate P, and CVH score was used as a continuous variable in the regression model.

Finally, to test the robustness and potential variations in different subgroups, we repeated all of the Cox proportional regressions stratified by age, gender, residence, ethnic group, marital status, education, alcohol consumption, previous CHD, and previous stroke groups, with CVH score as a continuous variable. Sensitivity analysis was performed: (1) excluding participants who had CHD or stroke previously; (2) excluding participants whose BMI is lower than 18 kg/m^2^; (3) excluding participants who have been followed up for less than 2 years; and (4) using propensity score weighted method with inverse probability of treatment weighting was adopted so as to test the robustness of results.

The statistical analyses were performed using SPSS 26.0 (SPSS Inc., IBM, Armonk, NY, USA) and R 4.1.2 (The R Foundation for Statistical Computing, Vienna, Austria; www.R-project.org). Two-sided *P*-values of < 0.05 were considered statistically significant.

## Results

### Baseline characteristics

The study sample consisted of 4,499 subjects, the baseline characteristics of whom are compared by the residence in Table 1. At the baseline survey, the average age of study population at baseline survey was 70.49(SD 6.77) years old, with 40.3% males and 98.1% Han nationality. Participants in urban areas had higher levels of education and morbidity than those in rural areas (CHD, hypertension, diabetes and dyslipidemia). Each crude CVH metric difference between residences was statistically significant (*P* < 0.001).

### CVH metrics and scores

**Fig. 1:**
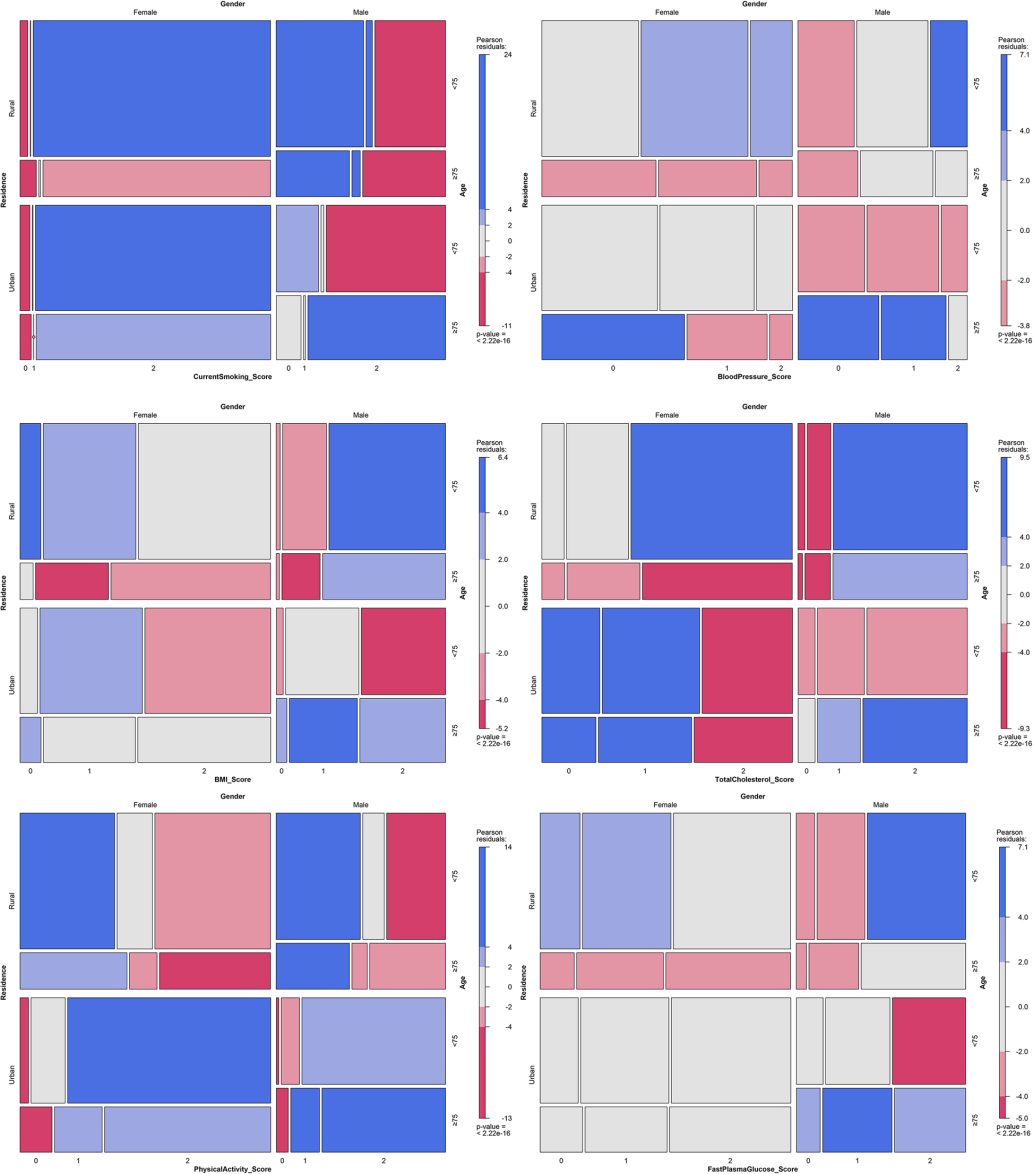
Mosaic plot of each ideal cardiovascular health metrics by age, gender and residence. Mosaic plot was drawn by R package “vcd” to depict the percentage of each ideal CVH in age and gender group. Female participants age < 75 years had the highest percentage of ideal CVH in smoking metric and male participants living in rural area did well in BMI metric. Compared with averaged expectation of ideal fasting plasma glucose, male participants who aged less than 75 years and lived in rural area had an excess percentage, while female participants who were at the same age and lived in urban area had less percentage

**Fig. 2:**
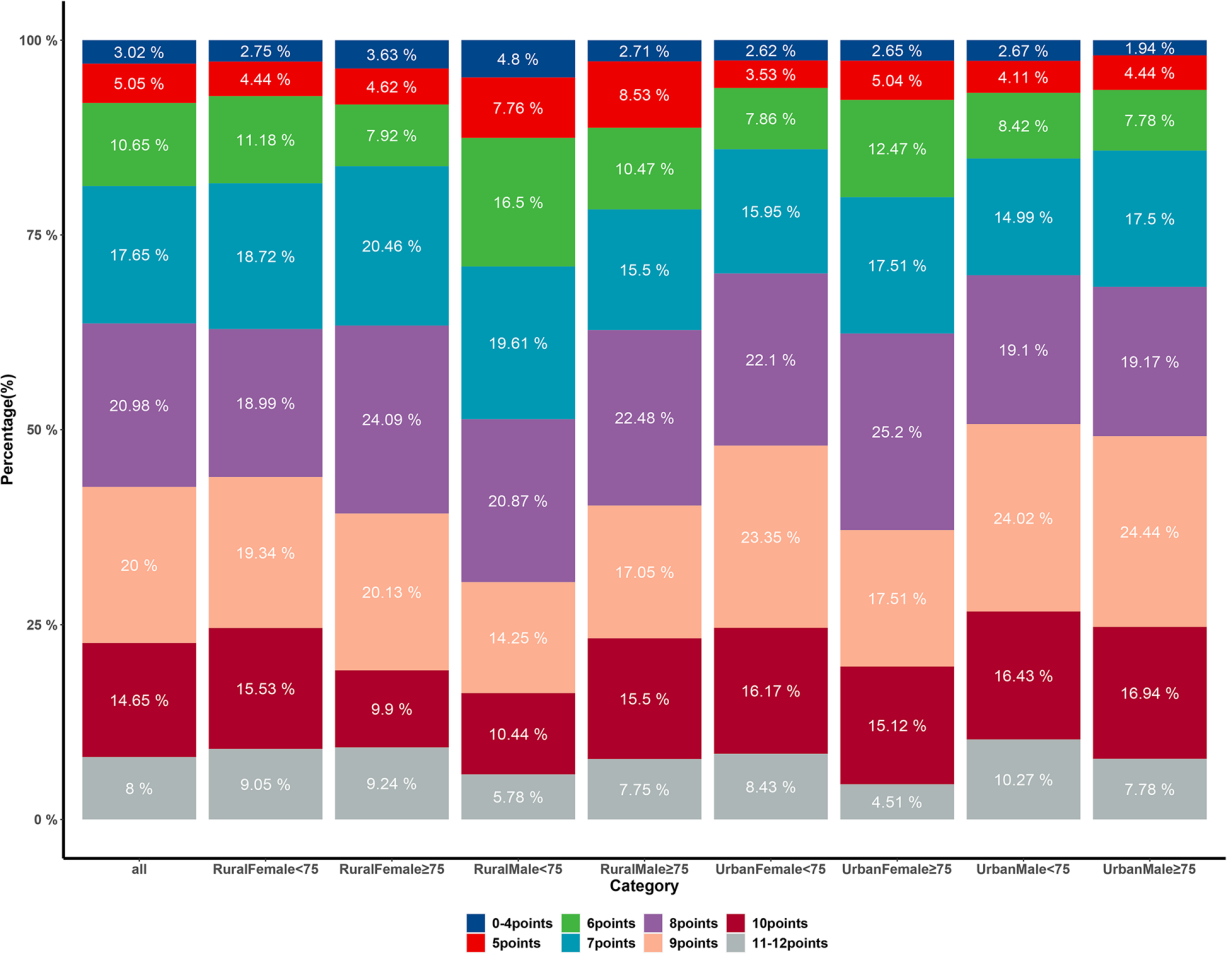
Percentage bar plot for the distribution of CVH scores by age, gender and residence. Percentage bar plot was drawn by R package “ggplot2” to present the distribution of CVH scores by the combination of age, gender and residence. It shows that only 8% study participants had 11–12 points (only 1.6% of participants obtained all the 6 metrics of ideal CVH) and the same centralized pattern was observed in subgroup

Table 2 shows the distribution of CVH metrics by gender, age, and residence group. Males had more ideal metrics than females (BMI, BP, TC, FPG), and urban residents had a higher rate of ideal CVH only in current smoking and physical activity. Figure 1 depicts additional subgroup combinations of ideal CVH metrics. In the smoking metric, female participants under the age of 75 had the highest ideal CVH percentage, while male participants living in rural areas performed well in the BMI metric. In comparison to the averaged expected value of ideal fasting plasma glucose, the proportion of male participants under 75 years old who lived in rural areas was relatively high, while the proportion of female participants under 75 years old who lived in urban areas was lower. Figure 2 depicts the distribution of CVH scores by subgroup. Only 8% of study participants had 11–12 points (only 1.6% had all six ideal CVH metrics), and the same centralized pattern was observed in each subgroup. The median CVH score in the study population was 8.00 (2.00). The CVH score was generally higher in participants who were under 75 years old and lower in participants who were over 75 years old.

**Table 2:** Distribution of cardiovascular health metrics and score in BECHCS^a^

Item	Overall	Gender		Age		Residence	
	N (%)	Male	Female	< 75	≥ 75	Urban	Rural
Current smoking			< 0.001		0.233		< 0.001
Yes	778(17.3)	671(37.0)	107(4.0)	572(17.9)	206(15.9)	231(11.0)	574(22.8)
Former, quit ≤ 12 months	58(1.3)	52(2.9)	6(0.2)	39(1.2)	19(1.5)	14(0.7)	44(1.8)
Never or quit > 12 months	3663(81.4)	1091(60.1)	2572(95.8)	2590(80.9)	1073(82.7)	1857(88.3)	1806(75.3)
Body mass index			< 0.001		0.068		< 0.001
≥ 30.0 kg/m^2^	270(6.0)	65(3.6)	205(7.6)	194(6.1)	76(5.9)	137(6.5)	133(5.5)
25.0–29.9 kg/m^2^	1637(36.4)	615(33.9)	1022(38.1)	1197(37.4)	440(33.9)	873(41.5)	764(31.9)
< 25.0 kg/m^2^	2592(57.6)	1134(62.5)	1458(54.3)	1810(56.5)	782(60.2)	1092(52.0)	1500(62.6)
Physical activity			0.005		0.351		< 0.001
Poor	1156(25.7)	511(28.2)	645(24.0)	834(26.1)	322(24.8)	112(5.3)	1044(43.6)
Intermediate	624(13.9)	233(12.8)	391(14.6)	430(13.4)	194(14.9)	312(14.8)	312(13.0)
Ideal	2719(60.4)	1070(59.0)	1649(61.4)	1937(60.5)	782(60.2)	1678(79.8)	1041(43.4)
Blood pressure			< 0.001		< 0.001		< 0.001
≥ 140 or ≥ 90 mmHg	1928(42.9)	711(39.2)	1217(45.3)	1297(40.5)	631(48.6)	1006(47.9)	922(38.5)
120–139 or 80-89 mmHg	1848(41.1)	776(42.8)	1072(39.9)	1348(42.1)	500(38.5)	815(38.8)	1033(43.1)
< 120 or < 90 mmHg	723(16.1)	327(18.0)	396(14.7)	556(17.4)	167(12.9)	281(13.4)	442(18.4)
Total cholesterol			< 0.001		0.781		< 0.001
≥ 240 mg/dL	544(12.1)	124(6.8)	420(15.6)	389(12.2)	155(11.9)	377(17.9)	167(7.0)
200-239 mg/dL	1239(27.5)	374(20.6)	865(32.2)	872(27.2)	367(28.3)	724(34.4)	515(21.5)
< 200 mg/dL	2716(60.4)	1316(72.5)	1400(52.1)	1940(60.6)	776(59.8)	1001(47.6)	1715(71.5)
Fasting plasma glucose			0.001		0.469		< 0.001
≥ 126 mg/dL	652(14.5)	226(12.5)	426(15.9)	477(14.9)	175(13.5)	332(15.8)	320(13.4)
100-125 mg/dL	1581(35.1)	627(34.6)	954(35.5)	1118(34.9)	463(35.7)	784(37.3)	797(33.2)
< 100 mg/dL	2266(50.4)	961(53.0)	1305(48.6)	1606(50.2)	660(50.8)	986(46.9)	1280(53.4)
CVH score, median (IQR)	8.00(2.00)	8.00(2.00)	8.00(2.00)	8.00(2.00)	8.00(2.00)	8.00(2.00)	8.00(2.00)
Behavior	5.00(2.00)	4.00(2.00)	5.00(2.00)	5.00(2.00)	5.00(2.00)	4.00(2.00)	5.00(2.00)
Factor	4.00(1.00)	4.00(2.00)	3.00(1.00)	4.00(2.00)	4.00(1.00)	4.00(2.00)	3.00(2.00)

^a^*BECHCS* Beijing elderly comprehensive health cohort study. χ^2^ method was conducted for categorical variables

### Association of CVH score with all-cause mortality

**Fig. 3:**
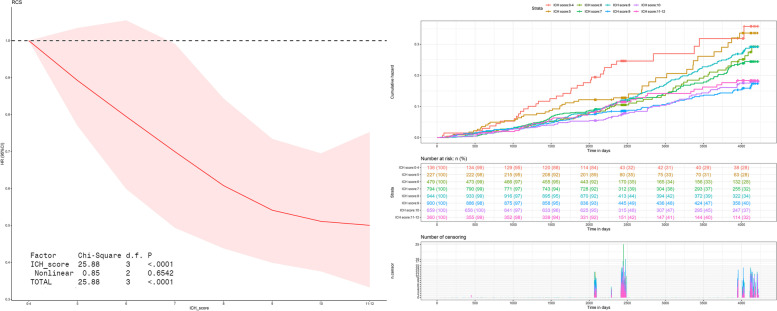
RCS and Survival curve for the association between CVH score category and all-cause mortality with unadjusted cox regression model. Restricted cubic spline was drawn by R package “rms” to test if the relationship between CVH score and all-cause mortality fits linear model. Survival curve was drawn by R package “survival” with cumulative hazard was generated to confirm whether CVH score fulfill the proportion hazard assumption. RCS: restricted cubic spline; ICH: ideal cardiovascular; The CVH score of 0–4 was set as the reference point with 4 knots. The result fits cox regression model

As Fig. 3 shows, the risk of all-cause mortality decreased linearly (P _nonlinear_ = 0.654) as the CVH score increased in restricted cubic spline (RCS) curve. In Supplemental Fig. 2, *P* values for nonlinear were 0.571, 0.570, and 0.575, respectively, for RCS curves with gradual adjustment. With a CVH score of 0–4 as a reference, each CVH score group was separated and the Cox regression assumption was followed.

There were 667 deaths among the study population during the average follow-up period of 8.13 years (36595.72 person-years). Subjects with a CVH score of 0–4 points had the highest rate and density of mortality [rate:24.26 (21.87,26.83); density: 33.02 (29.42,37.05) per 1,000 person years], while subjects with a CVH score of 11–12 points had a sharp decrease [rate: 13.33(12.17,14.59); density: 16.38 (14.87,18.03) per 1,000 person years]. In addition, as the CVH score increased, so did the survival rate.

Table 3 shows the association estimated by Cox regression. In all models with gradual adjustments, participants with a CVH score of 6 to 10 and 11–12 had a lower risk of all-cause mortality compared to those with a score of 0–4. After gradually adjusting the potential confounders in four models, participants with highest CVH score were associated with 52.0%, 51.0%, 44.6%, 41.6% reduced risk of all-cause mortality [model 1: HR = 0.480, 95%CI: (0.308,0.747); model 2: HR = 0.490, 95%CI: (0.314,0.763); model 3: HR = 0.554, 95%CI: (0.355,0.865); model 4: HR = 0.584, 95%CI: (0.373,0.913)] than that with lowest CVH score group.1.42%, 4.49%, 7.03%, 7.13%, 12.31%, 8.31% and 3.44% of the deaths could be avoided if the study population shifted to a CVH score ranged from 5 to 10 and 11–12, respectively. The HRs (95% CIs) of per point higher CVH score with gradual adjustment for all-cause mortality for continuous CVH score were 0.900 (0.863,0.939), 0.907 (0.870,0.947), 0.925 (0.886,0.965), and 0.928 (0.889,0.969), respectively.

**Table 3:** Cox proportional hazard model for the association of CVH with all-cause mortality

All-cause mortality	Mortality rate		Mortality density		Model 1^a^	Model 2^b^	Model 3^c^	Model 4^d^	PAF^d^, %
	Death/N	Percentage, %	n/person-year	per 1,000 person-years					
					HR (95%CI)				
Continuous	667/4499	14.83(14.47,15.19)	667/36595.72	18.23(17.76,18.70)	0.900(0.863,0.939)	0.907(0.870,0.947)	0.925(0.886,0.965)	0.928(0.889,0.969)	
0–4	33/136	24.26(21.87,26.83)	33/999.32	33.02(29.42,37.05)	Reference	Reference	Reference	Reference	-
5	42/227	18.50(16.83,20.30)	42/1710.66	24.55(22.15,27.20)	0.741(0.469,1.168)	0.668(0.423,1.054)	0.686(0.434,1.083)	0.723(0.457,1.143)	-1.42
6	72/479	15.03(13.96,16.17)	72/3714.11	19.39(17.92,20.97)	0.582(0.386,0.879)	0.559(0.370,0.844)	0.571(0.378,0.863)	0.596(0.394,0.902)	-4.49
7	121/794	15.24(14.40,16.12)	121/6314.21	19.16(18.03,20.36)	0.568(0.386,0.834)	0.559(0.381,0.822)	0.603(0.410,0.888)	0.628(0.427,0.925)	-7.03
8	166/944	17.58(16.76,18.44)	166/7678.70	21.62(20.53,22.77)	0.643(0.436,0.921)	0.610(0.420,0.887)	0.650(0.447,0.946)	0.683(0.468,0.995)	-7.13
9	107/900	11.88(11.18,12.64)	107/7644.53	14.00(13.12,14.93)	0.402(0.272,0.594)	0.395(0.267,0.584)	0.435(0.294,0.645)	0.452(0.304,0.670)	-12.31
10	78/659	11.84(11.01,12.71)	78/5603.05	13.92(12.90,15.02)	0.402(0.267,0.604)	0.395(0.262,0.594)	0.448(0.297,0.675)	0.476(0.315,0.720)	-8.31
11–12	48/360	13.33(12.17,14.59)	48/2931.13	16.38(14.87,18.03)	0.480(0.308,0.747)	0.490(0.314,0.763)	0.554(0.355,0.865)	0.584(0.373,0.913)	-3.44
P for trend	z = -2.103	*P* = 0.035	z = -2.351	*P* = 0.0187	< 0.001	< 0.001	< 0.001	0.001	

^a^Model 1: unadjusted crude model. Model 2: adjusted for age, gender, residence. Model 3: adjusted based on model 2 plus ethnic group, marital status, education, alcohol drinking. Model 4: adjusted based on model 3 plus previous coronary heart disease and previous stroke

^b^95% confidential intervals for mortality rate and density were based on Binomial Probabilities

^c^The value of P for trend was calculated by Mann–Kendall trend test for mortality rate and density. For the regression, P for trend was computed by including CVH score as continuous variable

^d^PAF: attributable fraction, $\mathrm{PAF}=\frac{\mathrm{pe}(\mathrm{HR}-1)}{1+\mathrm{pe}(\mathrm{HR}-1)}$, p_e_ refers to the proportion of exposed subjects in the population

### Subgroup and sensitivity analyses

**Fig. 4:**
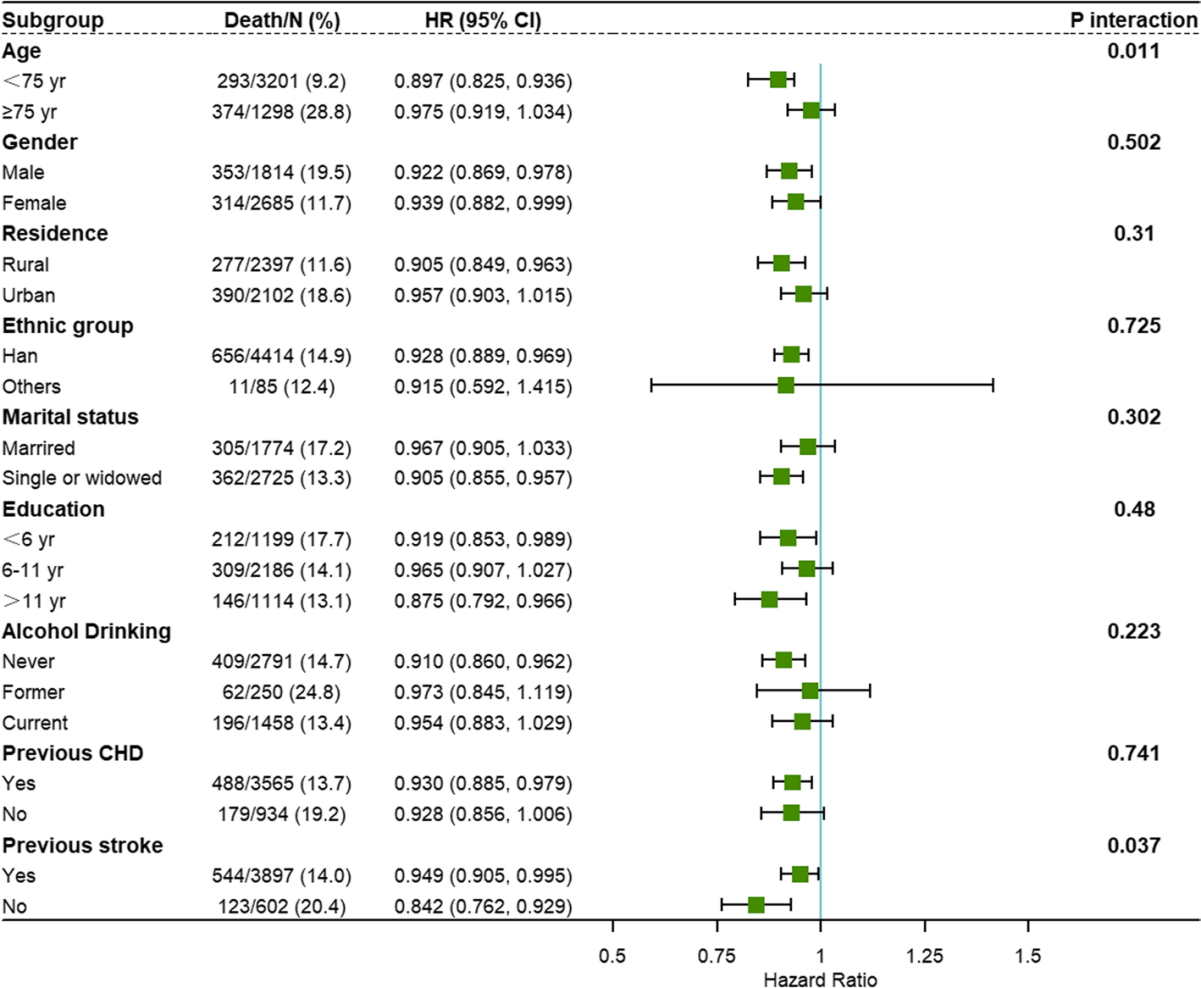
Subgroup analysis for the association of CVH with all-cause mortality. Forest plots were draw by R package “forestplot” to conduct subgroup analysis. Cox regression model adjusted for age, gender, residence, ethnic group, marital status, education, alcohol drinking, previous coronary heart disease and previous stroke. P for trend was computed by including CVH score as continuous variable. VH: cardiovascular health. In age-stratified analysis, each one-unit higher CVH score was similarly associated with a smaller likelihood of all-cause mortality among the participants aged under 75 years (*P* < 0.001). The association was similar across residence-stratified analysis, the increasing trend wasn’t observed (*P* = 0.146). In both male and female groups, the higher CVH score, the lower risk of all-cause mortality (P _male_ = 0.008, P _female_ = 0.042)

Figure 4 depicts forest plots for subgroup analysis to estimate the association of CVH score with all-cause mortality with full adjustments. Each one-unit higher CVH score was associated with lower all-cause mortality in younger adults(< 75 years old) compared to older adults (≥ 75 years old), and in participants with previous stroke compared to those without previous stroke (P for interaction = 0.011 and 0.037, respectively). Other subgroups' results were not significantly different. Given the power of model, we further adjusted the numbers of potential confounders (Supplemental Fig. 3). Sensitivity analyses were performed in Table 4 to test the robustness of the association. After excluding participants who had previously had CHD or stroke (1356), those with BMI less than 18 kg/m2 (100), and those who died within 2 years of follow-up (95), and using the propensity score weighting method for all participants individually, the results remained consistent.

**Table 4:** Sensitivity analysis for the association of CVH score with all-cause mortality

	Model 1^b^	Model 2^b^	Model 3^b^
Participants without prior CHD^a^ or stroke, n/total = 406/3143			
0–4, n/total = 15/79	Reference	Reference	Reference
5, n/total = 24/155	0.639(0.355,1.151)	0.566(0.314,1.02)	0.566(0.314,1.020)
6, n/total = 43/336	0.633(0.363,1.105)	0.606(0.347,1.059)	0.653(0.373,1.143)
7, n/total = 72/548	0.709(0.412,1.219)	0.634(0.368,1.092)	0.682(0.395,1.177)
8, n/total = 103/669	0.496(0.284,0.866)	0.478(0.273,0.837)	0.525(0.299,0.920)
9, n/total = 70/616	0.453(0.255,0.808)	0.417(0.233,0.745)	0.477(0.266,0.853)
10, n/total = 50/477	0.498(0.267,0.929)	0.495(0.265,0.925)	0.566(0.302,1.059)
11–12, n/total = 29/263	0.790(0.415,1.506)	0.639(0.335,1.219)	0.649(0.340,1.238)
P for trend^c^	0.001	0.004	0.044
Participants with BMI over 18 kg/m^2^, n/total = 645/4399			
0–4, n/total = 33/136	Reference	Reference	Reference
5, n/total = 42/225	0.585(0.388,0.884)	0.567(0.376,0.857)	0.607(0.401,0.918)
6, n/total = 72/475	0.569(0.387,0.836)	0.565(0.384,0.831)	0.636(0.432,0.937)
7, n/total = 120/784	0.618(0.425,0.900)	0.600(0.412,0.874)	0.675(0.462,0.985)
8, n/total = 159/915	0.389(0.263,0.576)	0.382(0.258,0.567)	0.437(0.294,0.650)
9, n/total = 103/886	0.399(0.265,0.601)	0.393(0.261,0.593)	0.476(0.314,0.721)
10, n/total = 75/636	0.426(0.269,0.673)	0.447(0.283,0.707)	0.539(0.340,0.856)
11–12, n/total = 41/342	0.744(0.472,1.175)	0.672(0.425,1.060)	0.729(0.461,1.153)
P for trend^c^	< 0.001	< 0.001	< 0.001
Participants with follow-up time over 2 years, n/total = 573/4404			
0–4, n/total = 28/131	Reference	Reference	Reference
5, n/total = 31/216	0.641(0.384,1.068)	0.573(0.344,0.956)	0.619(0.370,1.035)
6, n/total = 62/469	0.585(0.374,0.914)	0.554(0.354,0.865)	0.592(0.378,0.927)
7, n/total = 110/783	0.599(0.396,0.907)	0.579(0.382,0.878)	0.657(0.433,0.998)
8, n/total = 145/923	0.639(0.426,0.958)	0.603(0.402,0.905)	0.681(0.453,1.024)
9, n/total = 87/880	0.373(0.244,0.572)	0.356(0.232,0.546)	0.412(0.268,0.633)
10, n/total = 69/650	0.406(0.262,0.630)	0.389(0.251,0.605)	0.479(0.306,0.748)
11–12, n/total = 41/352	0.472(0.292,0.763)	0.478(0.296,0.773)	0.581(0.358,0.944)
P for trend^c^	< 0.001	< 0.001	< 0.001
All participants analyzed with PSW^d^, n/total = 667/4499			
0–4, n/total = 33/136	Reference	Reference	Reference
5, n/total = 42/227	0.740(0.466,1.176)	0.742(0.467,1.179)	0.739(0.465,1.175)
6, n/total = 72/479	0.579(0.381,0.880)	0.580(0.382,0.881)	0.580(0.382,0.881)
7, n/total = 121/794	0.568(0.384,0.842)	0.564(0.381,0.836)	0.564(0.381,0.836)
8, n/total = 166/944	0.634(0.433,0.928)	0.638(0.436,0.934)	0.640(0.437,0.938)
9, n/total = 107/900	0.403(0.271,0.601)	0.406(0.273,0.606)	0.408(0.274,0.609)
10, n/total = 78/659	0.401(0.265,0.606)	0.410(0.271,0.620)	0.412(0.272,0.624)
11–12, n/total = 48/360	0.481(0.306,0.756)	0.478(0.304,0.751)	0.483(0.307,0.760)
P for trend^c^	< 0.001	< 0.001	< 0.001

^a^CHD: coronary heart disease

^b^Model 1: unadjusted crude model. Model 2: adjusted for age, gender, residence. Model 3: adjusted based on model 2 plus ethnic group, marital status, education, alcohol drinking, previous coronary heart disease and stroke

^c^P for trend was computed by including CVH score as continuous variable

^d^PSW: propensity score weighted method; inverse probability of treatment weighting was applied for gradual adjustment of covariates. Model 1: age, gender and residence; Model 2: based on model 2 plus ethnic group, marital status, education and alcohol drinking; Model 3: based on model 3 plus previous coronary heart disease and stroke

## Discussion

This study investigated the relationship between CVH score with all-cause mortality among Chinese elderly: the higher the CVH score in old age, the lower the risk of all-cause mortality; and a linear-dose response relationship was observed. In both male and female population, consistent relationships were found between the obtainment of per point in CVH score and the reduced risk of mortality. This study provides evidence that CVH metrics can reduce the risk of all-cause mortality in the Chinese elderly population.

The prevalence of ideal CVH in Chinese elderly remains low. Our study found that 8% of the participants scored 11–12 points in CVH, and only 1.6% got all six metrics of ideal CVH. This result was consistent with a previous study [35], it included 5026 participants aged 65 and above in rural China, and found that only 0.8% of them met the ideal levels for 6 CVH metrics. Several studies have found a low prevalence of ideal CVH: 5% [36] (participants had ≥ 5 out of 7 metrics at the ideal level), 12% [37] (participants had 9–12 out of 12 points of CVH score), 6.5% [25] (participants had 5 to 7 health metrics at optimal levels). Due to the various definitions of ideal CVH, the distribution of CVH metrics components varies from one another. In our study, the low prevalence of ideal CVH resulted in the unfavorable blood pressure level: only 16.1% of all the subjects achieved the ideal state. It could be partially due to the use of biomass fuel [38] or a diet with high sodium [39]. In China, the hypertension is common, accounting for 33.0–47.2 of atherosclerotic CVD events [40], while the control rate is low [41]. Immediate actions are required in China to improve the prevention or treatment of hypertension in the elderly. Furthermore, because the score of metabolic CVH metrics would be affected by behavior metrics, composite metrics would be expected to be lower [20].

In this study, the CVH score was generally higher among the elderly men under 75 years old who live in the rural areas. Bi et al. investigated a nationally representative sample of 96,121 Chinese adults aged 20 years and older in 2010 and discovered that being female and being younger were the protective factors for cardiovascular health [42]. While Han et al. reported men were more likely than women to achieve ideal levels on all CVH metrics, smoking was not one of them [35]. Of note, smoking metric directed primarily at the sex difference in global and behavior CVH metrics, where males outnumber females in terms of smoking prevalence.. The same patterns were observed in urban and rural disparities. The inconsistency is likely due to the fact that premenopausal women are relatively unaffected by CVD risk, whereas sex disparity diminishes with age after menopause [43]. Previous studies found that the oldest old and centenarians had a higher overall level of CVH metrics than the young elderly [11, 34, 44, 45], but the prevalence of ideal CVH still needs to be improved. Furthermore, the difference between gender, age and residence in our study could be influenced by the population characteristics in our study as well as the sample size of subgroups. More related studies are favorable.

Strong associations of the CVH metrics with all-cause mortality have been confirmed. Our study found that the risk among Chinese elderly with CVH score at 11–12 points was 41.6% lower than that of those with a CVH score of 0–4 points (HR = 0.584, 95% CI: 0.373–0.913). As the number of CVH metrics increased, the risk of all-cause mortality decreased continuously and linearly[HR = 0.925, 85%CI: (0.885,0.967); (P _nonlinear_ = 0.575)]. In lined with our findings, Zhou et al. found that in the Chinese population, participants with higher ideal CVH metrics had a 54% lower risk of all-cause mortality compared to those with lower ideal metrics [32]. A stronger protective effect was observed in comparison to previous studies in younger adults [30–33]. The linear dose–response relationship was consistent with a meta-analysis [27], which included 142,137 participants to quantify the mortality risk of each unit change in ideal CVH and found that there is a linear inverse relationship. Previous studies [31, 46–48] recognized this close relationship, which were summarized in a meta-analysis [28], and the evidence was well established among younger populations. For older age adults, Gaye et al. conducted a Three-City Study to examine the benefit of higher CVH status in terms of mortality among participants aged 65 years and over [36]. The estimated HR (95% CI) was 0.71 (0.55–0.90) of all-cause mortality among subjects with ≥ 5 vs. ≤ 2 ideal metrics was assessed. Several studies in the Korean and British elderly population [34, 37] confirmed the evidence of an association between CVH and cardiovascular events in the elderly population. Then, our findings supplemented this gap among Chinese elderly even more. With the increase of ideal CVH score, the risk of all-cause mortality reduction was consistent across all age groups, highlighting the importance of promoting CVH metrics in old age. Furthermore, because the linear relationship was shown to be statistically significant, it should be emphasized that improving every point of CVH score- that is, shifting each CVH metric from poor into intermediate- systematically yielded favorable outcomes [18]. While achieving the ideal CVH is undoubtedly a long-term goal, the immediate action or short-term goal is to encourage the attainment of per point of CVH score at a time progressively [36].

Subgroup analysis could provide supporting evidence for more efficient methods of promoting CVH metrics in the elderly. In the age-stratified analysis, the decreasing trend in the risk of all-cause mortality with increasing CVH score was statistically significant among the younger elderly (age under 75 years), but not among the very elderly (age 75 years and above). The reason accounting for this could be “survivor bias”, which means that participants had to live to reach age 75 years old or older before the assessment of CVH score [49]. This shows the importance of paying special attention to the young and elderly when providing prevention or care. In the stroke-stratified analysis, more considerations are favorable to determine the difference of pattern of CVH metrics among older adults with stroke. In our study, individuals in rural areas tended to have higher levels of unhealthy lifestyles. According to current research [11, 18, 21], the disparity in health behavior between urban and rural areas is partly explained by socioeconomic development factors such as income and education level. This suggests that public health policies and strategies should be implemented especially focusing on behavior interventions for Chinese elderly in rural areas.

There are several limitations to the study. First, the prevalence of ideal CVH metrics in this study should be generalized with caution, given the non-nationally representative study population. The primary goal of our study was to evaluate the association of CVH score with all-cause mortality in the elderly. Further research into the epidemiology of CVH among the elderly in China would benefit from larger-scale or nationwide samples of the elderly. Second, data about lifestyles and diseases were self-reported, which could lead to recall bias. To address this problem, highly stringent training procedures and quality assurance programs were implemented throughout the research process. Including recruiting trained interviewees, requiring certificate of disease diagnosis, etc. if possible. Third, individuals with prior CVD were included in the analysis throughout, which might bring over estimation of the reported associations. To address this problem, sensitivity analysis was performed to exclude subjects with CHD or stroke, ensuring robustness of results. Besides, subjects with extremely low BMI or who died two years after follow-up were also excluded to account for misclassification bias. Fourth, detailed information was absent. To avoid classification bias and remain consist with previous study, we revised Life’s Simple 7 into 6 metrics. And because all of the data was measured once, any differences between the baseline survey and the endpoint investigation may have gone unnoticed. Furthermore, the risk of CVD mortality wasn’t estimated. Fifth, the sample sizes limited further stratified analysis or potential confounders. Considering the power of statistical analysis, we did gradual adjustments with 4 models, which might explain the reason why some associations were not statically significant.

## Conclusion

Among the Chinese elderly, there was a linear rekationship between CVH scores and the lower risk of all-cause mortality. It is critical to develop strategies to improve cardiovascular health attainment among Chinese elderly with low CVH scores.

## Supplementary Information

**Additional file 1:** **Supplemental Figure 1.** Participants enrolled and follow-up from September, 2009 to September, 2021 in BECHCS. **Supplemental Figure 2.** Restricted cubic spline for the association of CVH score with all-cause mortality. **Supplemental Figure 3.** Subgroup analysis for the association of CVH with all-cause mortality. **Supplemental Table 1.** Definition of 6 ideal cardiovascular health metrics according to American Heart Association*.

## Data Availability

Currently, the datasets used and/or analyzed during the current study are available from the corresponding author upon reasonable request.

## Abbreviations

CVD: Cardiovascular disease
CHD: Coronary heart disease
CVH: Cardiovascular health
BECHCS: Beijing elderly comprehensive health cohort study
HR: Hazard ratio
CI: Confidential interval
AHA: American heart association
BMI: Body mass index
