# Supplementary material for: Association of cardiovascular health at old age with all-cause mortality: a prospective cohort study in China
Source: BMC Geriatr. 2023 Jul 15;23:437. doi: 10.1186/s12877-023-04093-9 (PMC10349403; doi:10.1186/s12877-023-04093-9)
Supplement: Supplementary file 1 — Additional file 1: Supplemental Figure 1. Participants enrolled and follow-up from September, 2009 to September, 2021 in BECHCS. Supplemental Figure 2. Restricted cubic spline for the association of CVH score with all-cause mortality. Supplemental Figure 3. Subgroup analysis for the association of CVH with all-cause mortality. Supplemental Table 1. Definition of 6 ideal cardiovascular health metrics according to American Heart Association*. [file 12877_2023_4093_MOESM1_ESM.docx]

**ONLINE APPENDIX**

**Supplemental Figure 1 Participants enrolled and follow-up from September, 2009 to September, 2021 in BECHCS.** The flowchart was drawn by software “draw.io Diagrams”. BECHCS was a community-based cohort study conducted in Beijing and its surveyed areas consist of Wanshoulu community and Miyun district, which were selected as representatives of the geographic and economic characteristics of Beijing, China. A two-stage randomized cluster sampling method to enroll eligible subjects (aged 60 years and older, having lived in the local areas at least 12 months).

**Supplemental Figure 2 Restricted cubic spline for the association of CVH score with all-cause mortality.** Cox regression model (a) adjusted for age, gender and residence; (b) adjusted for age, gender, residence, ethnic group, marital status, education and alcohol drinking; (c) adjusted for age, gender, residence, ethnic group, marital status, education, alcohol drinking, previous coronary heart disease and previous stroke. The CVH score of 0-4 was set as the reference point with 4 knots. Each CVH score group was separated and followed the assumption of Cox regression.

**Supplemental Figure 3** S**ubgroup analysis for the association of CVH with all-cause mortality.** Cox regression model (a) adjusted for age, gender and residence; (b) adjusted for age, gender, residence, ethnic group, marital status, education and alcohol drinking; (c) adjusted for age, gender, residence, ethnic group, marital status, education, alcohol drinking, previous coronary heart disease and previous stroke. P for trend was computed by including CVH score as continuous variable. CVH: cardiovascular health. And the results were consistent with previous findings.

**Definition of the metrics of the Cardiovascular Health**

The definition of poor, intermediate and ideal metrics is provided in Table 1 and follows AHA criteria, except for the diet and physical activity metrics for which adaptations were required.

Supplemental Table 1 Definition of 6 ideal cardiovascular health metrics according to American Heart Association*

| Metric | | Poor Health | Intermediate Health | Ideal Health |
| --- | --- | --- | --- | --- |
| Current smoking ^a^ | | Yes | Former, quit≤12 months | Never or quit>12 months |
| Body mass index ^b^ | | BMI≥30.0 kg/m^2^ | 25.0 kg/m^2^≤BMI＜29.9 kg/m^2^ | BMI<25.0 kg/m^2^ |
| Physical activity ^c^ | | None | moderate or vigorous intensity≤4 hours/week | moderate or vigorous intensity≥5 hours/week |
| Blood pressure ^d^ | | Systolic≥140mmHg or  diastolic≥90mmHg | Systolic 120-139mmHg or diastolic 80-89mmHg, or treated to goal | Systolic<120mmHg and diastolic<90mmHg |
| Total cholesterol ^e^ | ≥6.20mmol/L(240mg/dL) | 5.17-6.19 mmol/L(200-239mg/dL) or treated to goal | ＜5.17mmol/L (200mg/dL) | |
| Fasting plasma glucose ^e^ | | ≥7.00mmol/L(126 mg/dL) | 5.56-6.99 mmol/L(100-125mg/dL) or treated to goal | <5.56mmol/L (100mg/dL) |

^a^ Smoking status was assessed using questions on current smoking status (current, ever, never) and duration of quitting smoking (years).

^b^ Body mass index equals to weight (kg) divide the square of height (m), which were measured by taking off shoes and wearing thin clothes with calibration limited to 0.5 kg/cm, and repeating the procedure twice for verification.

^c^ Physical activity was assessed by questions on the duration of participation in moderately energetic (walking, dancing, mild exercise like Tai Chi ) and vigorous physical activity (running, swimming, playing balls, cycling).

^d^ Blood pressure was measured twice with a sphygmomanometer in the sitting position after 5 min rest, and the average of the two readings was used in the present analyses.

^e^ Fasting blood glucose and total cholesterol were measured using standardized methods, and the conversion factors were 0.0556 and 0.0258, respectively.

*Dietary information was not available.


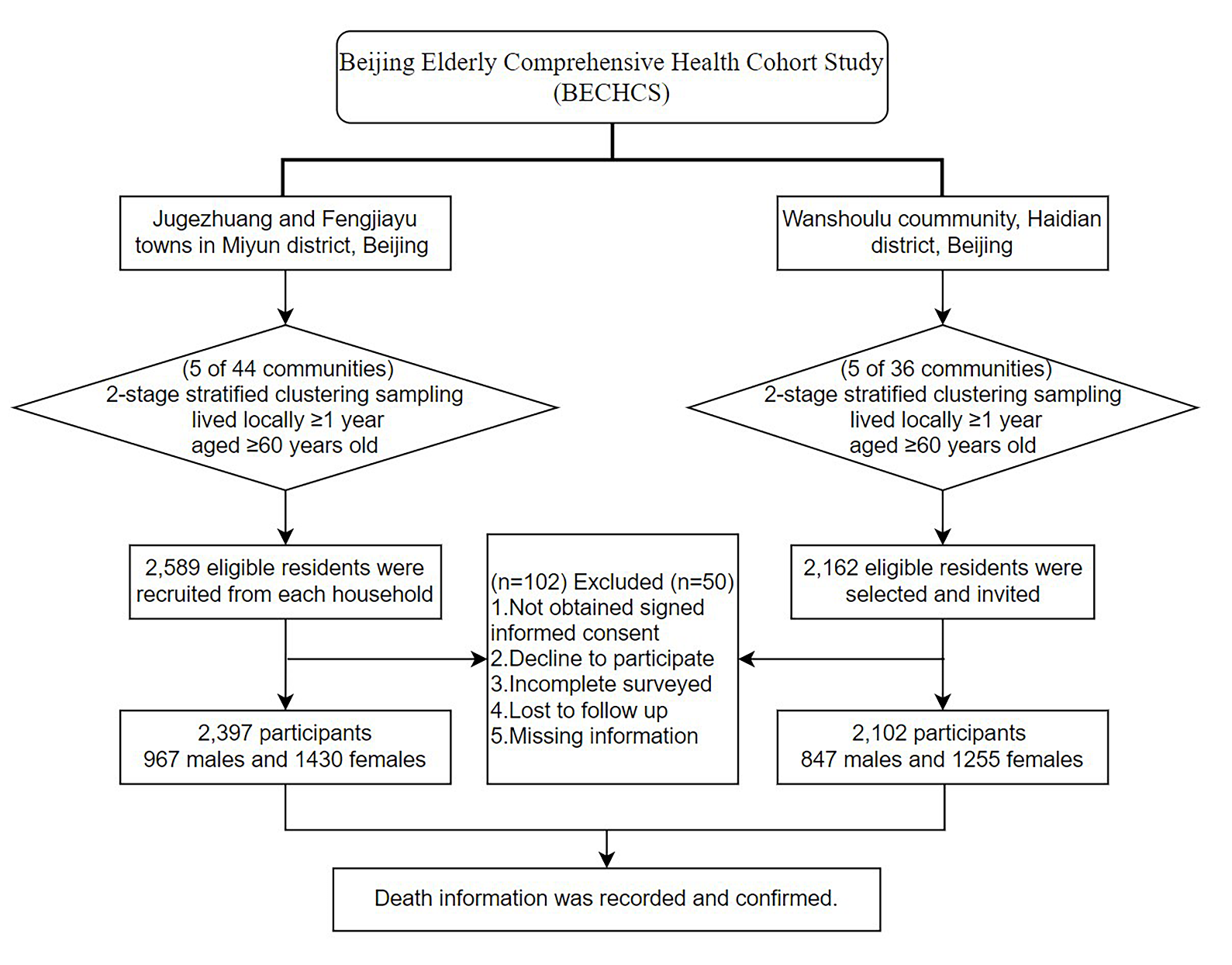
**Supplemental Figure 1 Participants enrolled and follow-up from September, 2009 to September, 2021 in BECHCS.**

**
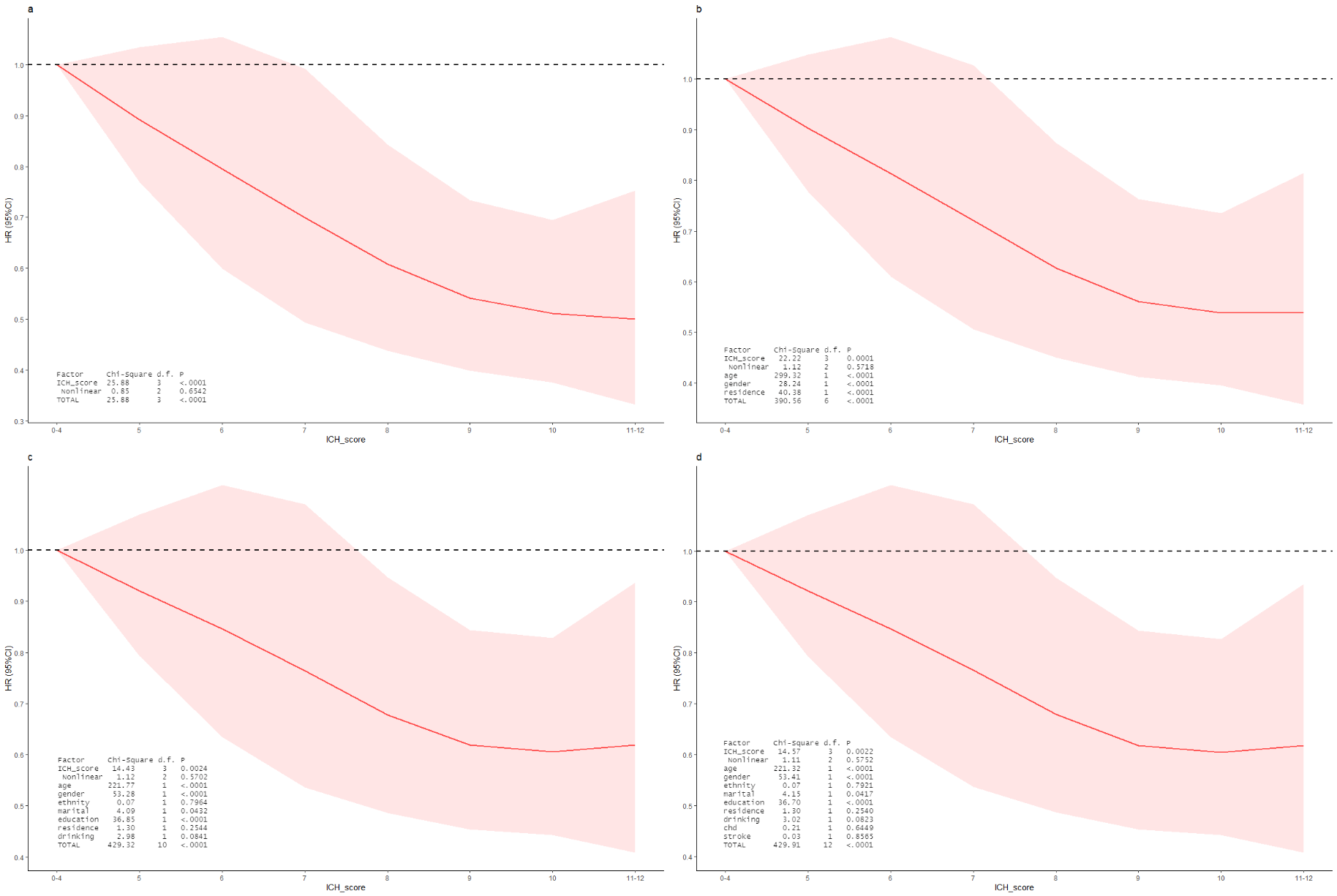
**

**Supplemental Figure 2 Restricted cubic spline for the association of CVH score with all-cause mortality.**


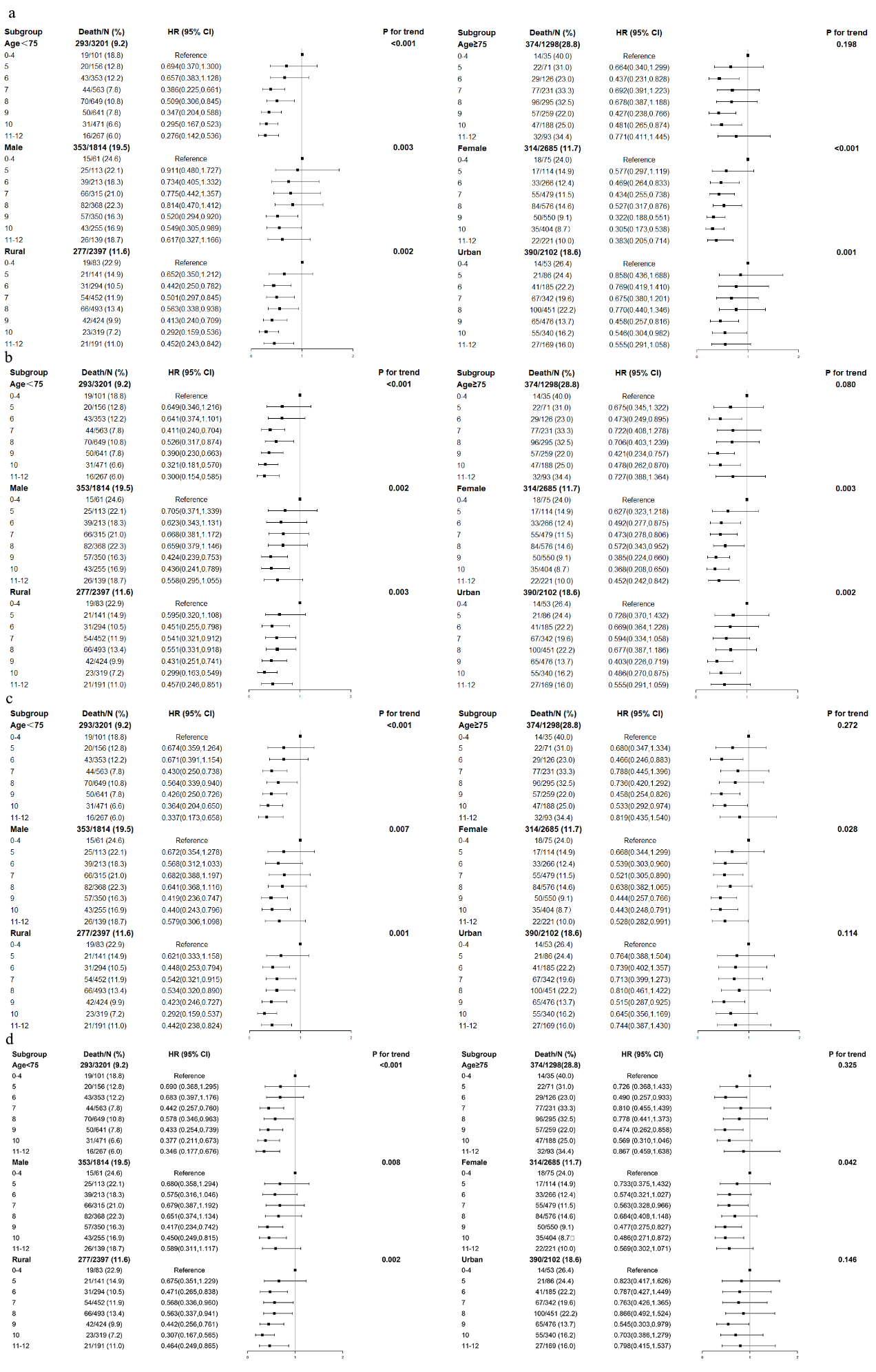


**Supplemental Figure 3** S**ubgroup analysis for the association of CVH with all-cause mortality.**
